# Supplementary material for: Fingerprints of brain disease: connectome identifiability in Alzheimer’s disease
Source: Commun Biol. 2024 Sep 18;7:1169. doi: 10.1038/s42003-024-06829-8 (PMC11411139; doi:10.1038/s42003-024-06829-8)
Supplement: Supplementary file 3 — Reporting Summary [file 42003_2024_6829_MOESM3_ESM.pdf]

nature portfolio

Corresponding author(s): Sara STAMPACCHIA, Enrico AMICO

Last updated by author(s): Jul 18, 2024

# Reporting Summary

Nature Portfolio wishes to improve the reproducibility of the work that we publish. This form provides structure for consistency and transparency in reporting. For further information on Nature Portfolio policies, see our Editorial Policies and the Editorial Policy Checklist.

## Statistics

For all statistical analyses, confirm that the following items are present in the figure legend, table legend, main text, or Methods section.

| n/a | Confirmed | |
|---|---|---|
| ☐ | ☒ | The exact sample size ($n$) for each experimental group/condition, given as a discrete number and unit of measurement |
| ☐ | ☒ | A statement on whether measurements were taken from distinct samples or whether the same sample was measured repeatedly |
| ☐ | ☒ | The statistical test(s) used AND whether they are one- or two-sided<br>*Only common tests should be described solely by name; describe more complex techniques in the Methods section.* |
| ☐ | ☒ | A description of all covariates tested |
| ☐ | ☒ | A description of any assumptions or corrections, such as tests of normality and adjustment for multiple comparisons |
| ☐ | ☒ | A full description of the statistical parameters including central tendency (e.g. means) or other basic estimates (e.g. regression coefficient) AND variation (e.g. standard deviation) or associated estimates of uncertainty (e.g. confidence intervals) |
| ☐ | ☒ | For null hypothesis testing, the test statistic (e.g. $F$, $t$, $r$) with confidence intervals, effect sizes, degrees of freedom and $P$ value noted<br>*Give P values as exact values whenever suitable.* |
| ☒ | ☐ | For Bayesian analysis, information on the choice of priors and Markov chain Monte Carlo settings |
| ☒ | ☐ | For hierarchical and complex designs, identification of the appropriate level for tests and full reporting of outcomes |
| ☐ | ☒ | Estimates of effect sizes (e.g. Cohen's $d$, Pearson's $r$), indicating how they were calculated |

*Our web collection on statistics for biologists contains articles on many of the points above.*

## Software and code

Policy information about availability of computer code

| | |
|---|---|
| Data collection | No software was used to acquire the data. The data for ADNI dataset is supplied by the publicly available databases Alzheimer's Disease Neuroimaging Initiative (ADNI) platform. The data for Geneva dataset was acquired in the Geneva University Hospital using a 3T Siemens Magnetom Skyra scanner. |
| Data analysis | Custom-code was implemented in Matlab R2022a for fingerprinrint analyses and in R Studio 2022.07.2 for statistical analyses. The full code necessary to reproduce the main results and figures of the work will be available online after publication in a maintained GitHub repository (github.com/ss1913/fingerprints_alzheimer). |

For manuscripts utilizing custom algorithms or software that are central to the research but not yet described in published literature, software must be made available to editors and reviewers. We strongly encourage code deposition in a community repository (e.g. GitHub). See the Nature Portfolio guidelines for submitting code & software for further information.

# Data

Policy information about availability of data

All manuscripts must include a data availability statement. This statement should provide the following information, where applicable:

- Accession codes, unique identifiers, or web links for publicly available datasets
- A description of any restrictions on data availability
- For clinical datasets or third party data, please ensure that the statement adheres to our policy

*Provide your data availability statement here.*

# Human research participants

Policy information about studies involving human research participants and Sex and Gender in Research.

| | |
|---|---|
| Reporting on sex and gender | Participants' sex was assigned at birth and was not based on self-report. Sex-based analyses were not conducted due to the insufficient sample size for meaningful subgroup analysis. There were no significant differences in the number of females and males within each group, so findings should be considered applicable to both sexes. While these results suggest generalizability, further research with a larger sample size would be beneficial to confirm these findings across both sexes. |
| Population characteristics | Covariate relevant population characteristics included sex, age, years of education, global cognitive functioning and amyloid load. |
| Recruitment | Participants were included from two independent cohorts: the Geneva Memory Centre (Geneva University Hospitals, Geneva, Switzerland) and the Alzheimer's Disease Neuroimaging Initiative (ADNI), using inclusion and exclusion criteria specified below. |
| Ethics oversight | The ADNI data used in this work belong to a publicly available database. All methods were carried out in accordance with relevant guidelines and ethical regulations and have been previously described (detailed information are provided in the manuscript). The study protocol for the Geneva dataset was approved by Commission Cantonale d'Ethique de la Recherche sur l'être humain (CCER) of the Geneva Canton (Switzerland). |

Note that full information on the approval of the study protocol must also be provided in the manuscript.

# Field-specific reporting

Please select the one below that is the best fit for your research. If you are not sure, read the appropriate sections before making your selection.

☒ Life sciences ☐ Behavioural & social sciences ☐ Ecological, evolutionary & environmental sciences

For a reference copy of the document with all sections, see nature.com/documents/nr-reporting-summary-flat.pdf

# Life sciences study design

All studies must disclose on these points even when the disclosure is negative.

| | |
|---|---|
| Sample size | Participants were included from two independent cohorts: the Geneva Memory Centre (Geneva University Hospitals, Geneva, Switzerland) and the Alzheimer's Disease Neuroimaging Initiative (ADNI). Inclusion criteria were availability of (i) fMRI and T1-weighted scans, (ii) 18F-Florbetapir or 18F-Florbetaben amyloid-PET to derive amyloid β-status (iii) clinical and cognitive assessments and demographic data, and (iv) identical fMRI acquisition parameters. Subjects ranged from healthy ageing and Aβ-negative (cognitively unimpaired, CU Aβ-), to mild cognitive impairment Aβ-positive (MCI Aβ+), and Aβ-positive subject with dementia due to probable AD, AD dementia. This resulted in N=58 participants for the Geneva cohort, and N=79 for the ADNI cohort. |
| Data exclusions | Exclusion criteria was the presence of any other significant neurologic disease than Alzheimer's Disease. These included: Parkinson's disease, multi-infarct dementia, Huntington's disease, normal pressure hydrocephalus, brain tumour, progressive supranuclear palsy, seizure disorder, subdural hematoma, multiple sclerosis, or history of significant head trauma followed by persistent neurologic deficits or known structural brain abnormalities. |
| Replication | Two replication datasets were included to ensure results' reproducibility and assess the impact of dataset-specific factors such as clinical (Geneva) vs. research datasets (ADNI). |
| Randomization | Clinical status (i.e., CU vs. MCI vs. Dementia) was established by expert neurologists of the Geneva Memory Centre for the Geneva cohort, and from ADNI collaborators for the ADNI cohort. Aβ-status was determined in two ways: using the previously established cut-points and/or visually determined by an expert nuclear medicine physician using visual assessment and standard operating procedures approved from the European Medicines Agency. |
| Blinding | Investigators were blind to group allocation. |

# Reporting for specific materials, systems and methods

We require information from authors about some types of materials, experimental systems and methods used in many studies. Here, indicate whether each material, system or method listed is relevant to your study. If you are not sure if a list item applies to your research, read the appropriate section before selecting a response.

## Materials & experimental systems

| n/a | Involved in the study |
| --- | --- |
| ☒ | ☐ Antibodies |
| ☒ | ☐ Eukaryotic cell lines |
| ☒ | ☐ Palaeontology and archaeology |
| ☒ | ☐ Animals and other organisms |
| ☐ | ☒ Clinical data |
| ☒ | ☐ Dual use research of concern |

## Methods

| n/a | Involved in the study |
| --- | --- |
| ☒ | ☐ ChIP-seq |
| ☒ | ☐ Flow cytometry |
| ☐ | ☒ MRI-based neuroimaging |

# Clinical data

Policy information about clinical studies

All manuscripts should comply with the ICMJE guidelines for publication of clinical research and a completed CONSORT checklist must be included with all submissions.

| | |
| --- | --- |
| Clinical trial registration | N/A |
| Study protocol | N/A |
| Data collection | N/A |
| Outcomes | N/A |

# Magnetic resonance imaging

## Experimental design

| | |
| --- | --- |
| Design type | Resting-state fMRI |
| Design specifications | Resting-state fMRI data were acquired in one run of approximately 7 (Geneva) and 8 (ADNI) minutes. |
| Behavioral performance measures | No behavioral performance measures were collected inside the MRI. |

## Acquisition

Imaging type(s): functional, anatomical

Field strength: 3T

Sequence & imaging parameters:

- Geneva: Structural and functional data were acquired using a 3T Siemens Magnetom Skyra scanner (Siemens Healthineers, Erlangen, Germany) using a 64-channels phased-array head coil. Scans were performed within the radiology and neuroradiology division, Geneva University Hospitals, Geneva, Switzerland. An EPI-BOLD sequence was used to collect functional data from 35 interleaved slices (slice thickness=3mm; multi-slice mode=interleaved; FoV=192x192x105mm; voxel size=3mm isotropic; TR=2000ms, TE=30ms; flip-angle=90°; GRAPPA acceleration factor=2, time points=200, approximate acquisition time=7 minutes). Whole-brain T1-weighted anatomical images were acquired using a 3D MPRAGE sequence (slice thickness=0.9mm; FoV=263x350x350mm; voxel size=1mm isotropic; TR=1930ms; TE=2.36ms, flip-angle=8 °; GRAPPA acceleration factor=3).

- ADNI, data was obtained using 3T MRI scanners with a standardised protocol across imaging sites (full details in https://adni.loni.usc.edu/wp-content/uploads/2017/07/ADNI3-MRI-protocols.pdf). An EPI-BOLD sequence was used to acquire functional data (slice thickness=3.4mm, FoV=220x220x163mm, voxel size=3.4 isotropic; TR=3000ms; TE=30ms; flip angle=90°; GRAPPA acceleration factor=2; time points =197, approximate acquisition time=10 minutes). Whole-brain T1-weighted anatomical images were acquired with a 3D MPRAGE sequence (slice thickness=1mm, FoV=208x240x256mm; voxel size=1x1x1mm; TR=2300ms, TE=3ms, flip angle=9°, GRAPPA acceleration factor=3).

Area of acquisition: Whole brain

Diffusion MRI: ☐ Used ☒ Not used

## Preprocessing

| | |
|---|---|
| Preprocessing software | fMRI data were preprocessed using in-house MATLAB code |
| Normalization | The parcellation in MNI coordinates was first normalised to the individuals' previously registered T1 image and then resampled to the lower functional BOLD resolution. Final analyses were in native space. |
| Normalization template | Parcellation was in Montreal Neurological Institute (MNI) standard space |
| Noise and artifact removal | Image preprocessing pipelines for the two cohorts included substantially similar steps.These included: T1-weighted volumes were skull-stripped, segmented into white, grey matter and cerebrospinal fluid and spatially coregistered to fMRI native space. fMRI volumes were corrected for nuisance signals including motion signals, average white matter and cerebrospinal fluid signals, and band-pass filtered to the band 0.01-0.15 Hz. |
| Volume censoring | No volume censoring was applied. |

## Statistical modeling & inference

| | |
|---|---|
| Model type and settings | Group-comparisons were performed using Chi-square test for sex, and one-way ANOVA or its non-parametric equivalent, i.e., Kruskal-Wallis test for age, years of education, cognitive score and amyloid load. In addition, we used one-way ANOVAs to test the effect of group on whole-brain fingerprint measures (ISelf and IOthers separately) after checking for nuisance variables, with 5000 permutations to control for sample size differences. Finally, a null model was specifically designed for our analyses: this involved shuffling of subjects' assignment to group labels to derive between-groups significance of nodal brain fingerprint. |
| Effect(s) tested | Matching the three groups for demographic and clinical data. Differences across the three groups in terms of whole-brain fingerprint measures. Identify nodes whose functional connectivity with the rest of the brain could account for significant differences in subject variability across the three groups. |

Specify type of analysis: ☒ Whole brain ☐ ROI-based ☐ Both

| | |
|---|---|
| Statistic type for inference (See Eklund et al. 2016) | None, not relevant. |
| Correction | None, not relevant. |

## Models & analysis

| n/a | Involved in the study |
|---|---|
| ☐ | ☒ Functional and/or effective connectivity |
| ☐ | ☒ Graph analysis |
| ☒ | ☐ Multivariate modeling or predictive analysis |

| | |
|---|---|
| Functional and/or effective connectivity | We estimated individual FC matrices using Pearson's correlation coefficient between the averaged signals of all regions pairs. |
| Graph analysis | Functional connectome graph analysis, weighted undirected graph, at the individual and group level. Statistical analysis of group differences at the edge level, nodal strength analysis at the individual and group level |
